# Divergent Resilience of Bacterial and Fungal Gut Microbiota After Colorectal Surgery: Insights From a Prospective Longitudinal Cohort Study

**DOI:** 10.1002/mco2.70781

**Published:** 2026-05-26

**Authors:** Simon Wetzel, Eva Kohnert, Roman Huber, Alexander Müller, Agnes Knott, Lampros Kousoulas, Clemens Kreutz, Mohamed Tarek Badr, Ann‐Kathrin Lederer

**Affiliations:** ^1^ Institute of Medical Microbiology and Hygiene, Faculty of Medicine, Medical Centre ‐ University of Freiburg University of Freiburg Freiburg Germany; ^2^ Centre For Inherited Metabolic Diseases (CMMS) Karolinska University Hospital Solna Sweden; ^3^ Department For Medical Biochemistry and Biophysics Karolinska Institute Stockholm Sweden; ^4^ Institute of Medical Biometry and Statistics, Faculty of Medicine and Medical Centre University of Freiburg Freiburg Germany; ^5^ Centre For Complementary Medicine, Department of Medicine II, Medical Centre ‐ University of Freiburg, Faculty of Medicine University of Freiburg Freiburg Germany; ^6^ Department of General and Visceral Surgery, Medical Centre ‐ University of Freiburg, Faculty of Medicine University of Freiburg Freiburg Germany; ^7^ Institute of Medical Microbiology and Hospital Hygiene, Medical Faculty Otto Von Guericke University Magdeburg Magdeburg Germany; ^8^ Research Group Integrative Medicine, Department of General and Visceral Surgery University Hospital Ulm Ulm Germany

**Keywords:** antimicrobial resistance, interkingdom interactions, KEGG, microbiome, perioperative medicine, surgery

## Abstract

The composition of the gut microbiota changes throughout life and is shaped by various external influences, particularly major physiological stressors such as surgery. The extent of these changes and their impact remain poorly understood. This prospective cohort study aimed to investigate changes in the gut microbiota following colorectal surgery and to identify factors that modify these alterations. Paired pre‐ and postoperative stool samples from 59 patients at the University Medical Centre Freiburg were analyzed using 16S rRNA and ITS2 gene sequencing. Analyses included alpha and beta diversity, LEfSe differential feature analysis, network analysis with Louvain clustering, KEGG pathway annotation, and correlation with clinical parameters. Bacterial diversity significantly decreased postoperatively (Shannon index: *p* < 0.001), while fungal diversity remained largely unchanged (p > 0.05). Beta diversity revealed increased inter‐patient variability in bacterial communities after surgery (PERMANOVA *p* = 0.001). Preoperative network analyses identified 18 microbial network clusters and interkingdom associations between bacteria and fungi. KEGG pathway mapping showed cluster‐specific metabolic profiles, including enrichment in degradation pathways, antimicrobial resistance mechanisms, and bacterial secretion systems. The contrasting responses of bacterial and fungal communities highlight the importance of considering the entire gut microbiome in perioperative care and suggest a central role for interkingdom interactions in maintaining gut homeostasis during surgical recovery.

## Graphical abstract

Patients undergoing colorectal surgery (*n* = 59) were sampled before and after surgery to investigate perioperative microbiome dynamics. Using 16S rRNA and ITS2 genes amplicon sequencing, bacterial diversity significantly decreased postoperatively, while fungal communities remained resilient. Integrated interkingdom network analysis identified 18 distinct bacterial‐fungal clusters, highlighting surgery‐associated shifts in microbial interactions.

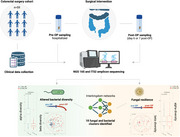

## 1 Introduction

The human body hosts a complex and symbiotic community of microorganisms, collectively known as the microbiota, which are vital for physiological functions [1, 2, 3]. Throughout life, each person develops an individual gastrointestinal profile of microorganisms, which is less diverse in infancy with a transition during childhood and adolescence and development of the full range of microorganisms in adulthood [4, 5]. The composition of the gut microbiota depends on the host‐related factors such as genetics, geographic or ethnic background, as well as diet and personal habits [5, 6, 7, 8]. The gut microbiota consists not only of bacteria but also other microorganisms, including fungi. It has been demonstrated that the gastrointestinal microbiota is of importance for the development and progression of diseases [5, 9]. The body's regular microbial inhabitants can lead to serious infections in certain situations, for example, *Escherichia coli* can cause urinary tract infections as well as life‐threatening conditions such as sepsis and meningitis [10, 11, 12]. Recent research suggests that the composition of the gut microbiota plays a role in human immunocompetence, as emphasized by studies on the immune response in patients with COVID‐19 [13].

Although microbial diversity is generally highly stable and resilient, it can be permanently disrupted by severe external influences [1, 4, 14, 15, 16]. Surgical procedures impose significant physiological and psychological stress on the body, profoundly affecting homeostasis and immune response [17, 18, 19]. During the perioperative period, multiple factors such as altered intestinal permeability, reduced gut motility, exposure to antibiotics, and nutritional changes may disrupt gut microbial communities. In modern perioperative practice, combined bowel preparation, consisting of mechanical cleansing and preoperative oral antibiotics, is recommended by the new German Perioperative Management of Gastrointestinal Tumours (POMGAT) guidelines [20]. It is suggested that preoperative oral antibiotic therapy can significantly influence the gut microbiota composition, although the extent and duration of these effects remain incompletely understood. Despite the well‐documented effects of surgical stress on overall host physiology, systematic investigations of perioperative gut microbiota changes are scarce and largely limited to the bacterial component [21, 22, 23]. Multiple factors have the capacity to induce alterations in the composition of the gut microbiota following surgical intervention [24]. During surgery, antibiotics are administered as part of wound infection prophylaxis. Postoperatively, patients’ mobility, diet, and environmental exposure differ from their usual habits due to the hospital stay [25, 26]. Patients may also receive a variety of drugs that may influence the gut microbiota after surgery [24].

Recent research has begun to explore the potential role of gut microbiota in influencing postoperative outcomes, including complications and recovery trajectories [8, 19, 22, 27]. These findings suggest that microbial community function, rather than taxonomy alone, may be a key determinant of clinical outcomes. Functional pathways involved in drug metabolism, epithelial barrier maintenance, and immune modulation are particularly relevant in the surgical context, where medication efficacy, wound healing, and infection risk represent primary clinical concerns. However, comprehensive analyses examining both bacterial and fungal components are notably lacking. This current gap is significant, as interkingdom interactions between bacteria and fungi may play crucial roles in maintaining gut homeostasis during physiological stress and in restoring this homeostasis post‐stress. For example, *Pseudomonas aeruginosa* can exert antagonistic effects on *Candida albicans*, whereas synergistic effects have been described for *Candida albicans* and *Staphylococcus aureus* [28, 29, 30]. The probiotic yeast *Saccharomyces boulardii* can modulate *Clostridioides difficile* toxin activity [31]. Other forms of bacterial‐fungal interactions include metabolic cross‐feeding, co‐biofilm formation, and modulation of antimicrobial compound production [32]. However, despite these well‐documented interactions in experimental systems, interkingdom dynamics in surgical patients remain largely unexplored, which underscores the importance of our study.

It is of high relevance to understand the extent to which the gut microbiota changes after surgery. It is also necessary to clarify which factors influence these alterations in order to develop evidence‐based strategies for maintaining gut homeostasis during surgical recovery. This prospective longitudinal study aims to (1) characterize postoperative alterations in bacterial and fungal gut microbiota following colorectal surgery, (2) identify interkingdom network interactions and their relationship to microbiome resilience, (3) perform functional pathway analysis to determine possible clinical implications of observed changes, and (4) characterize clinical factors influencing these perioperative microbiome dynamics. Our primary hypothesis was that bacteria and fungi respond differently to surgical stress and thereby alter gut homeostasis.

## 2 Results

**FIGURE 1:**
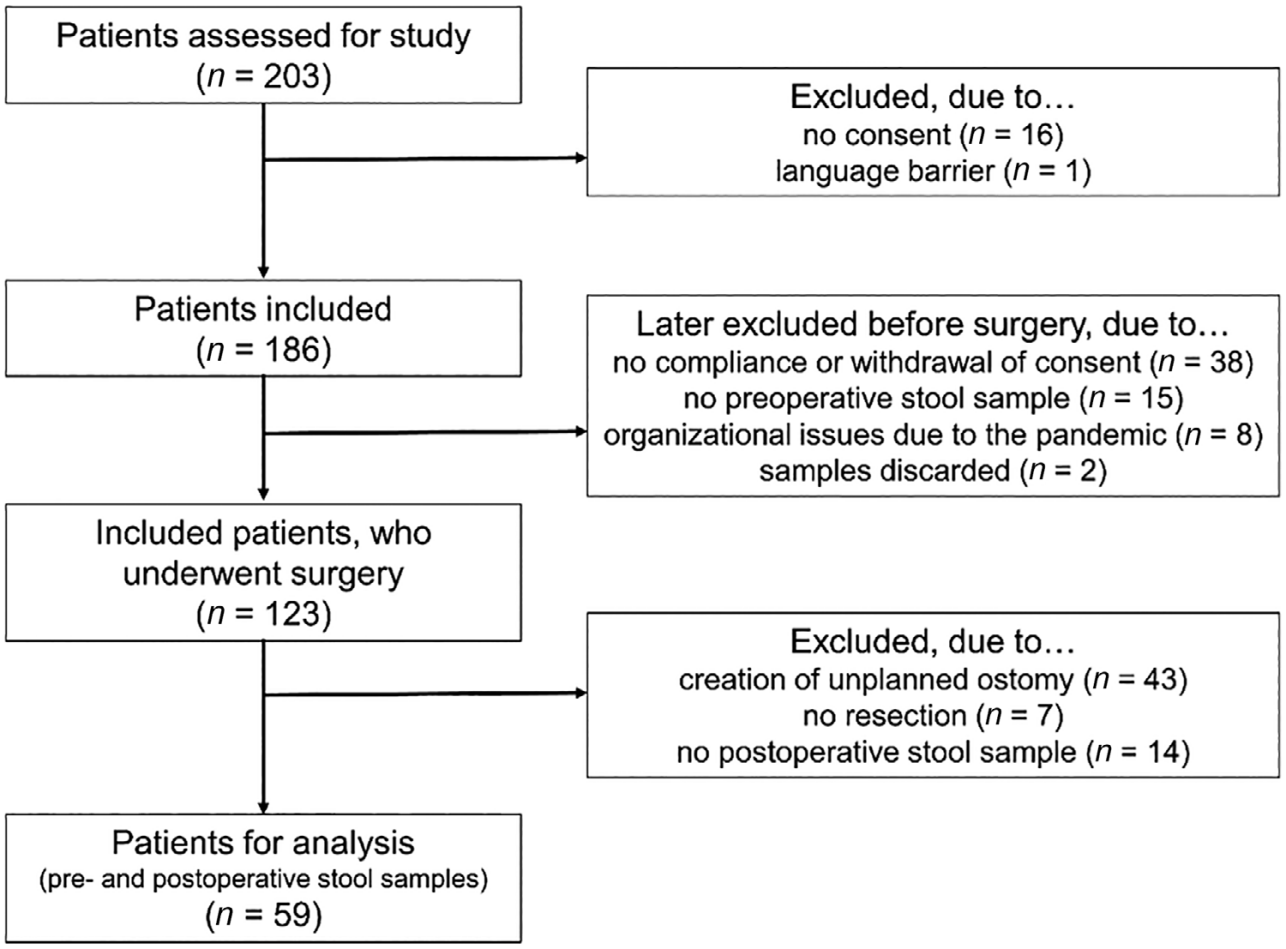
Patient recruitment flowchart and study participation. Flowchart illustrating patient screening, enrolment, and exclusion process. From initially 203 screened patients, more than a quarter of eligible patients (27%) did not consent to participate (*n* = 16), withdrew consent or had no compliance (*n* = 38). A further 15 patients declared that they wanted to continue participating in the study but did not provide a preoperative stool sample in time. Two preoperative stool samples were discarded by third parties. In 43 patients, the surgical approach was changed during the operation, resulting in the creation of a transient or permanent ostomy. Fourteen patients did not provide a postoperative stool sample. A major challenge was the outbreak of the COVID‐19 pandemic in 2021, which ultimately led to the discontinuation of further recruitment. Eight patients who had been scheduled to participate could not be included due to hygiene regulations that prohibited non‐medically necessary contact with patients. Out of 203 patients who were preoperatively screened for study participation, 59 patients with complete pre‐ and postoperative stool samples were ultimately included in final analysis. The flowchart was created using Microsoft PowerPoint.

Overall, 203 patients were preoperatively screened for evaluation. An overview of the whole recruitment process is shown in Figure 1. 59 patients with complete pre‐ and postoperative stool sample pairs were ultimately included for evaluation.

The average age of the analyzed patients was 62.4 ± 10.6 years. Almost half of the patients were male (*n* = 27, 46%). On average, the patients were of normal weight and were classified as between 2 and 3 according to the physical status classification system of the American Society of Anesthesiologists. Complete baseline characteristics are shown in Table 1.

**TABLE 1:** Overview of patients’ characteristics. (ASA = American Society of Anesthesiologists, SD = standard deviation, *up to 4 weeks before surgery).

Parameter	Value ± SD (range)
Age (years)	62.4 ± 10.6 (36‐83)
ASA physical status classification	2.58 ± 0.6 (2‐4)
Body Mass Index (kg/m^2^)	24.8 ± 4.6 (18‐38)
Pack‐years (years)	9.36 ± 14.9 (0‐60)
	***n* (%) / *n* (%)**
Sex (male/female)	27 (46%) / 32 (54%)
Surgical approach (open/laparoscopic)	24 (41%) / 35 (59%)
Antibiotics before surgery* (yes/no)	8 (14%) / 51 (86%)
Diet (omnivorous/vegetarian)	57 (97%) / 2 (3 %)
	***n* (%)**
**Comorbidities and pre‐existing disease**	
Cancer previously	36 (61%)
Cancer currently	34 (58%)
Cardiovascular	27 (46%)
Diabetes	7 (12%)
Chronic renal failure	7 (12%)
**Concomitant medication**	
Antihypertensive	31 (53%)
Inhibition of platelet aggregation	13 (22%)
Thyroid medication	12 (20%)
Anticoagulants	8 (14%)
Cholesterol reducing drugs	8 (14%)
Immunosuppression	2 (3%)
Steroids	1 (2%)
**Performed surgery**	
Right hemicolectomy	27 (46%)
Sigmoid resection	15 (25%)
Continuity restoration	10 (17%)
Left hemicolectomy	5 (9%)
Other	2 (3%)
**Previous surgery**	
Never	19 (32%)
Non‐abdominal	17 (29%)
Abdominal	13 (22%)
Both	10 (17%)
**Smoking**	
Never	34 (58%)
Previously	15 (25%)
Active	10 (17%)
**Alcohol consumption**	
Occasionally	38 (63%)
No	18 (31%)
Regularly	4 (7%)
**Drug abuse**	
No	57 (97%)
Cannabis	2 (3%)

Next, we analyzed the bacterial and fungal gut microbiome of all included patients in this study by bacterial 16S and fungal ITS2 targeted next‐generation sequencing (NGS) of collected pre‐ and postoperative fecal samples. High‐quality sequencing data were obtained for all samples. Bacterial *16S rRNA* gene sequencing yielded a mean of 41,223 reads per sample after quality filtering (range: 2234–105,773). Fungal ITS2 sequencing produced on average 41,818 reads per sample after initial filtering (range: 588–419,035). Rarefaction curves reached saturation for both bacterial and fungal communities and low base calling error rates were achieved, indicating adequate sequencing depth and quality (Figure S1).

### 2.1 Surgical Intervention Impacts Bacterial Gut Diversity and Community Structure

**FIGURE 2:**
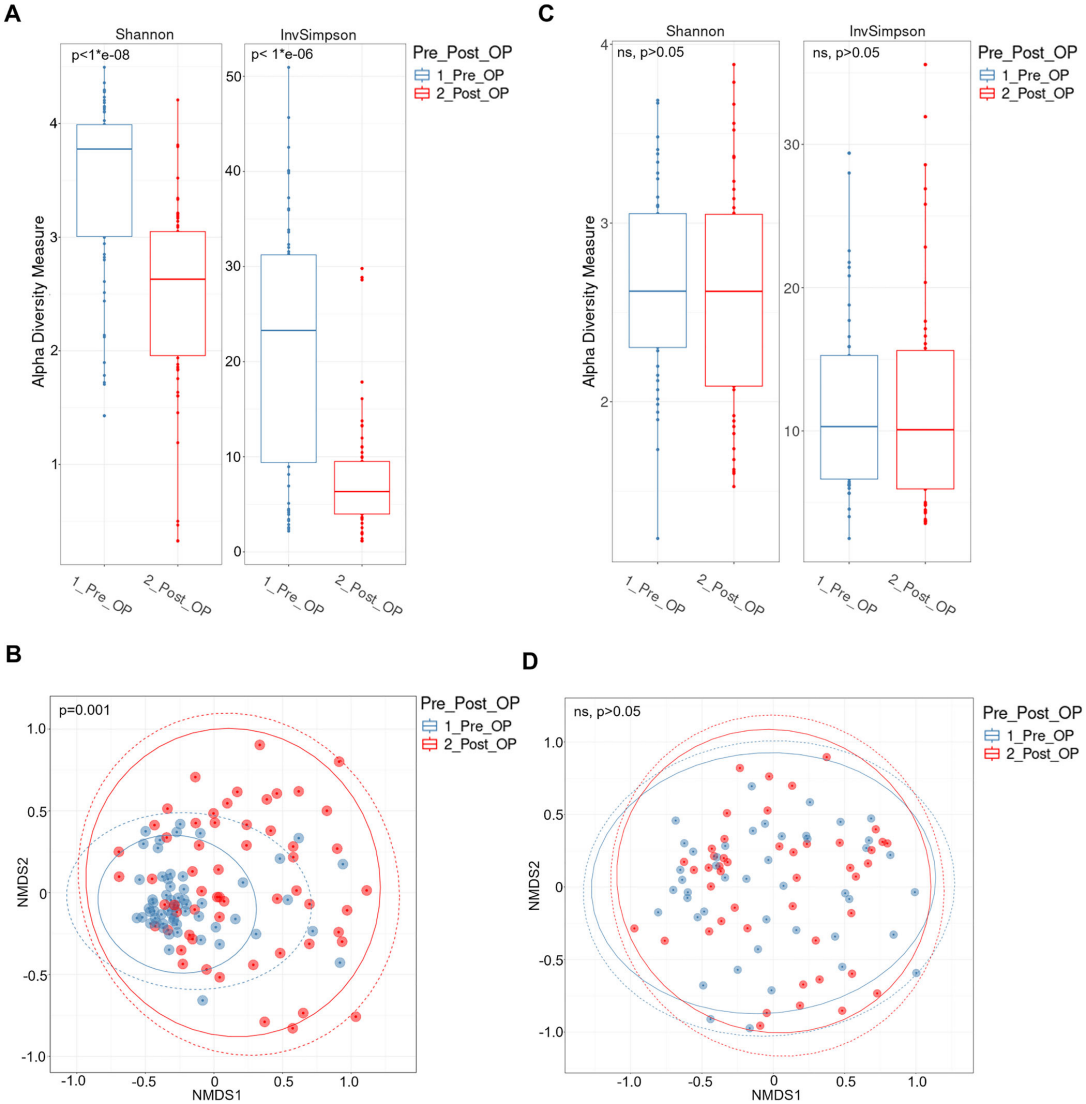
Bacterial and fungal gut alterations pre‐ and postoperatively. (A) Bacterial alpha diversity in patients before and after colorectal surgery measured by Shannon and Inverse Simpson indices. (B) Bacterial beta diversity based on Bray–Curtis dissimilarity distances. Each point represents an individual patient sample (red = preoperative, blue = postoperative). (C) Fungal alpha diversity in patients before and after colorectal surgery measured by Shannon and Inverse Simpson indices. (D) Fungal community structure visualized by Non‐metric Multidimensional Scaling (NMDS) ordination based on Bray–Curtis dissimilarity distances. Each point represents an individual patient sample (red = preoperative, blue = postoperative). Pre_OP = preoperative, Post_OP = postoperative, g = taxonomic genus level. Boxplots display median values (horizontal line), interquartile ranges (boxes), and data distribution (whiskers and points). The figure was generated using R.

We observed a significant decrease in alpha diversity within the bacterial gut microbiome of patients undergoing bowel surgery on the fifth to sixth postoperative day compared to the preoperative microbiome (*p* < 0.001, Figure 2A). While the bacterial gut microbiota composition was highly similar between patients preoperatively, bacterial beta diversity was found to be significantly increased postoperatively (*p* = 0.001), indicating diverging gut alterations between individual patients after surgical intervention (Figure 2B). We did not identify any significant drivers for these diverging effects by correlation analysis with comorbidities, clinical outcomes or the resected colon part (see Figure S2A–D). Overall bacterial alpha and beta diversity was not significantly different between females and males.

Based on these initial findings, we hypothesized that the overall postoperative decrease of bacterial gut alpha diversity accompanied by an increased beta diversity in this cohort is indicative of a postoperatively less diverse and more dysbiotic microbiome with an increase in potentially pathogenic bacterial species in response to surgical stress and perioperative antibiotic treatment. To test this hypothesis, we analyzed perioperative changes of the bacterial microbiome on phylum, genus and species levels. We did not observe relevant abundance changes of bacteria on high taxonomic ranks between pre‐ and postoperative samples, i.e., bacterial phylum level (Figure S2E). By in‐depth abundance analysis, employing the LefSe analysis classification tool, we identified 23 bacterial genera that were assigned to either pre‐ or postoperative state with a linear discriminant analysis (LDA) effect size >3 (*p* > 0.05), reflecting differential abundance trends in preoperative and postoperative stool samples (Figure S3). Notably, preoperative samples showed higher relative abundances of beneficial microbiota including *Faecalibacterium* and *Bifidobacterium* genera, which are associated with gut health and anti‐inflammatory functions [33, 34]. Conversely, postoperative samples demonstrated significant increases in potential opportunistic pathogens including *Klebsiella* and *Enterobacter*, genera commonly associated with hospital‐acquired infections and soft tissue complications. Other health‐associated bacteria that decreased postoperatively included *Coprococcus* and *Lactobacillus*.

### 2.2 Mycobiome Dynamics in Response to Colorectal Surgery With Unchanged Overall Diversity

In contrast to the effect seen in the bacterial communities, fungal alpha diversity was not significantly changed on the fifth to sixth postoperative day (Figure 2C). Additionally, no significant fungal beta diversity changes were observed between pre‐ and postoperative samples (Figure 2D), indicative of a primarily unaffected fungal diversity in patients after colorectal surgery. Despite this overall unchanged diversity in the entire cohort, we observed different fungal diversity trends in subgroups: patients undergoing colorectal resection (80%) exhibited lower fungal diversity after surgery, whereas continuity restoration surgery (17%) was associated with higher postoperative fungal diversity measurements assessed by the Shannon index (p>0.05, Figure S4). These contrasting patterns may suggest surgery‐specific effects on the mycobiome.

### 2.3 Bacteriome‐Mycobiome Interactions and Microbiota Clusters in the Preoperative Microbiome

**FIGURE 3:**
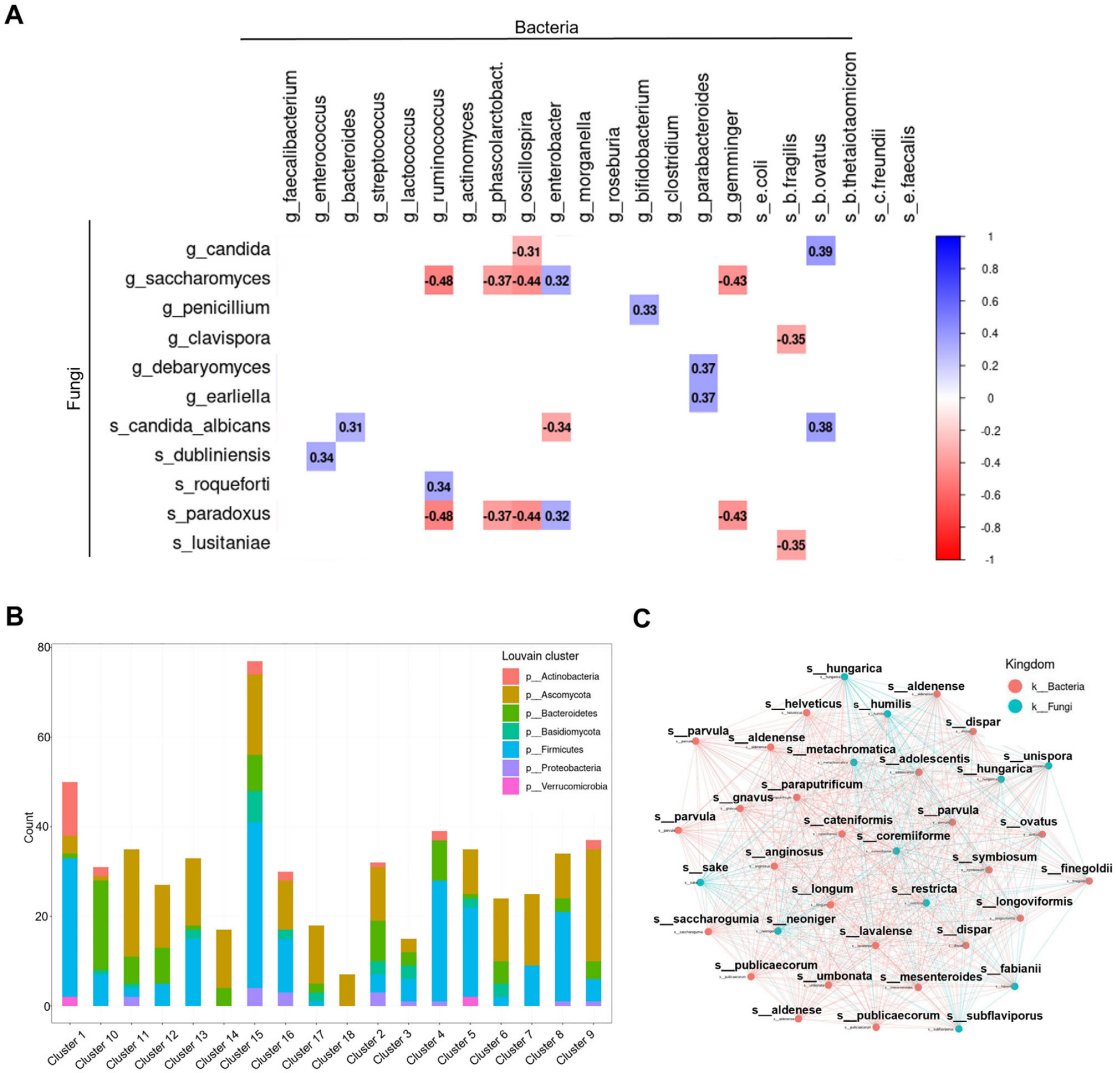
Bacteriome and Mycobiome network analysis and interkingdom cluster identification of the preoperative microbiome. (A) Heatmap of fungal‐bacterial species interactions in the preoperative gut microbiome. Colors represent the strength and direction of Spearman correlations (blue = positive, red = negative), with intensity indicating correlation magnitude. Only statistically significant correlations (*p* < 0.05) are displayed with correlation coefficients. (B) Distribution of identified microbial clusters at taxonomic genus level. Colored bars represent the composition on phylum level and relative abundance of each cluster (1–18). (C) Visualization of the central microbiota network hub with high connectivity (degree value >50). Nodes represent individual microbial species (red = bacterial, blue = fungal), with connecting lines indicating significant interactions. The figure was generated using R.

As part of a more holistic clinically‐driven approach, considering both the natural complexity of the human gut microbiome and NGS data, we aimed to identify outcome‐relevant alterations of fungal and bacterial microbiota in the context of colorectal surgery. First, we investigated species‐specific associations between fungi and bacteria by analyzing presurgical 16S and ITS2 NGS data using statistical correlation analysis (Figure 3A). We identified 22 associations between fungi and bacteria, either indicating a symbiotic co‐abundance in ten cases or potential competitive mechanisms in twelve cases. Based on these results, we performed bacteriome‐mycobiome network analysis using SpiecEasi R package and Louvain clustering of presurgical NGS data, revealing interkingdom network interactions and cluster formation of bacterial and fungal gut microbiota (Figure S5A). In total, 18 microbial clusters were identified, 16 of which were of both bacterial and fungal origin (Figure S5C). Most of the bacterial and fungal Amplicon Sequent Variants (ASVs) detected in these clusters belonged to fungal *Ascomycot*a (including *Candida*, *Saccharomyces* and *Aspergillus* genera) or bacterial *Firmicutes* (like *Clostridia* and *Lactobacilli* genera) on the phylum level (Figure 3B), in line with their natural abundance in the human gut. We further performed a degree distribution analysis of the complete microbial network showing a strong increase of interactivity toward its center (Figure S5B) and revealing a cluster of 36 closely associated fungal (11/36, 31%) and bacterial (25/36, 69%) species with a network degree value above 50 (Figure 3C).

### 2.4 Clinical Factors Influence Microbial Network Structure

#### 2.4.1 Patient Characteristic Correlations

**FIGURE 4:**
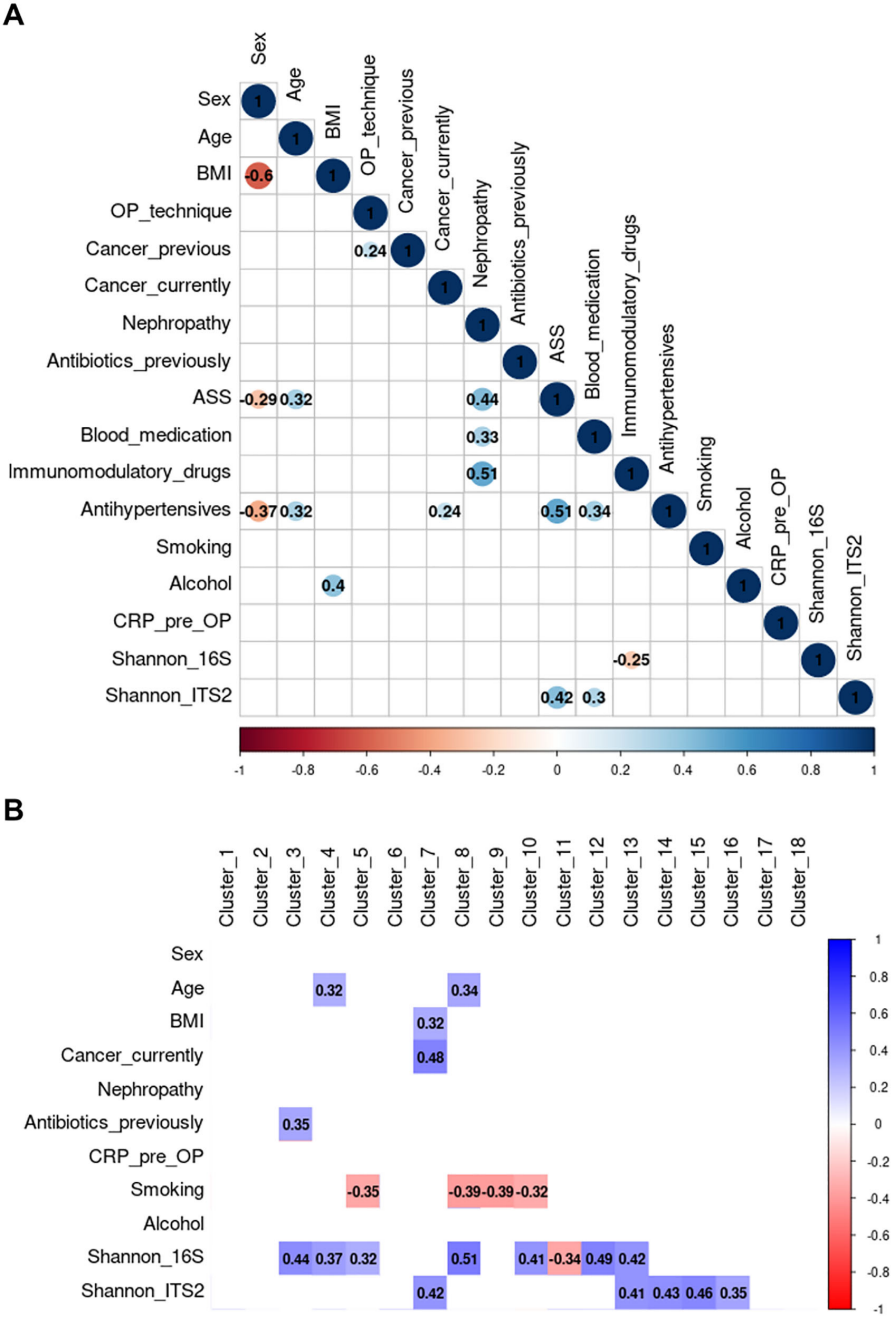
Clinical correlations and microbiota cluster associations. (A) Correlation heatmap of clinical metadata with preoperative microbial diversity metrics. The color intensity indicates the strength and direction of Spearman correlations, with blue representing positive correlations and red representing negative correlations. Only statistically significant correlations (*p* < 0.05) are displayed with correlation coefficients. (B) Heatmap showing associations between identified microbial clusters (columns) and clinical parameters or diversity metrics (rows). Color intensity represents the strength and direction of correlations, with blue indicating positive and red indicating negative associations. Numbers represent correlation coefficients for significant associations (*p* < 0.05). The figure was generated using R.

To investigate other factors influencing microbiota dynamics and possible clinical confounders, we performed statistical correlation analysis with collected clinical data. Initially, we further assessed cohort characteristics by analyzing possible associations between different clinical parameters, i.e., demographical data (sex, age, body mass index [BMI]), comorbidities, medication, presurgical inflammatory markers, and nicotine/alcohol consumption (Figure 4A). Gender‐specific differences were observed as females presented with a lower BMI than males [*p*_corr_<0.001, *r* = ‐0.60] and were less commonly treated with antihypertensives [*p*_corr_<0.05, *r* = ‐0.37], possibly implying a better cardiovascular status of females on the cohort level. Further, regular and occasional alcohol consumption was associated with a higher BMI [*p*_corr_<0.05, *r* = 0.40]. Next, we correlated clinical data with preoperative 16S and ITS2 NGS gut microbiome data. Surprisingly, preoperative bacterial or fungal alpha diversity was not significantly affected by preoperative antibiotic treatment (4‐week period) nor strongly associated with any clinical characteristics or preoperative markers. We did not detect significant associations between the collected clinical data and relative abundances of individual microbial genera or species in the preoperative gut.

#### 2.4.2 Cluster‐clinical Associations

We performed preoperative cluster integration with clinical data and assessed cluster‐associated diversity measures (Figure 4B). Microbiota abundances of nine clusters (cluster 2–10) showed significant associations with different patient characteristics (age, BMI, antibiotic treatment, smoking). Smoking negatively affected abundances of four identified microbiota clusters, of which cluster 5, 8 and 10 were associated with higher preoperative bacterial diversity. Furthermore, six of the identified clusters (cluster 11–16) showed significant associations with preoperative bacterial or fungal diversity and were not significantly associated with any other patient metadata, implying a direct association unaffected by considered possible confounders. Notably, a single fungal and bacterial cluster (cluster 11), correlated with lower preoperative bacterial diversity, mainly consisting of ASVs belonging to *Clostridium perfringens*, *Saccharomyces paradoxus*, *Penicillium roqueforti*, *Candida albicans*, *Bacteroides ovatus*, and *Clostridium perfringens* species. A higher abundance of the central network cluster (cluster 15) was not associated with bacterial diversity measures, however cluster abundance significantly correlated with patients’ fungal diversity.

### 2.5 Cluster and Functional Analysis Reveal Clinically Relevant Metabolic Signatures

**FIGURE 5:**
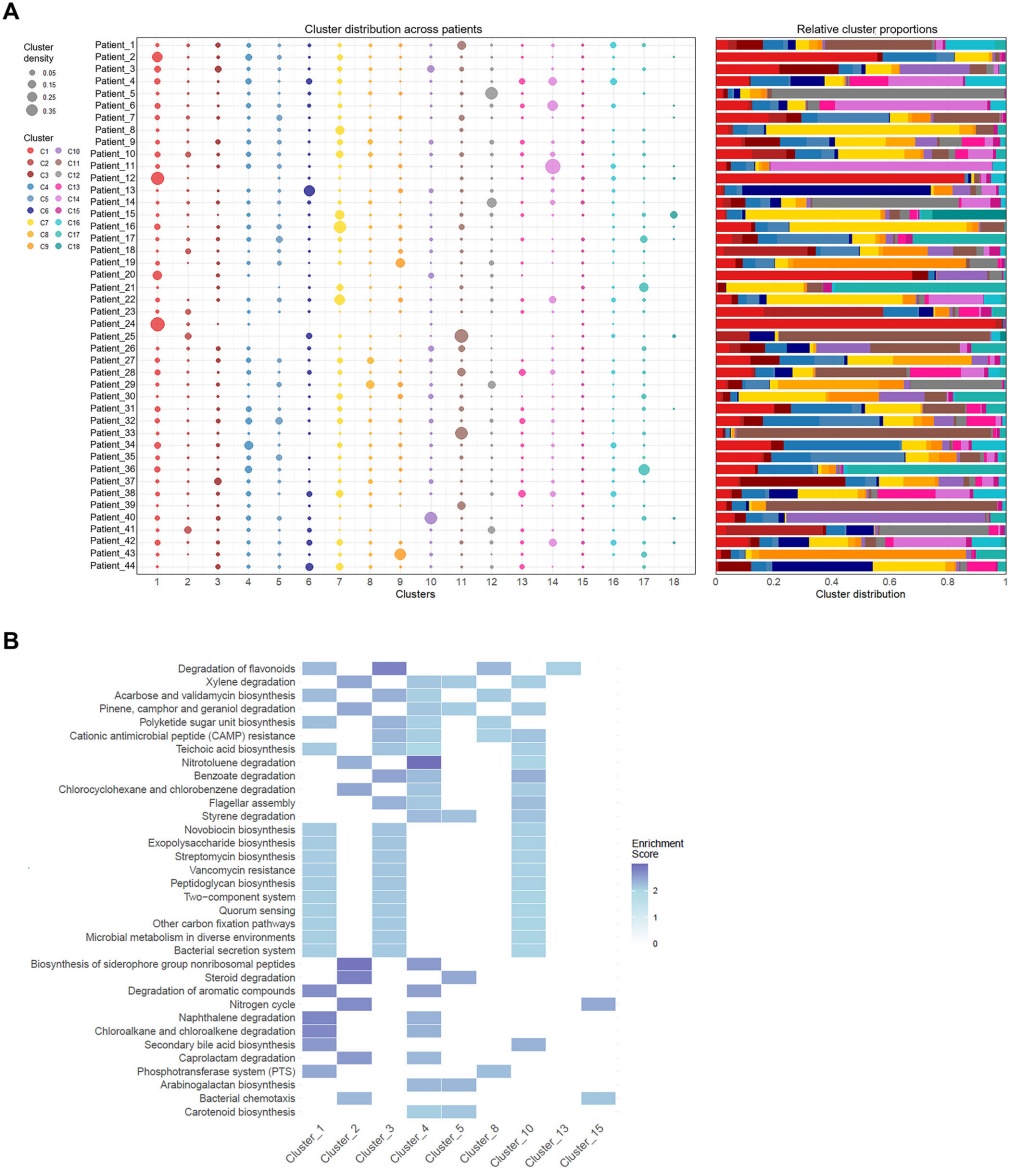
Comprehensive cluster‐based analysis of preoperative microbiome community structure and functional metabolic mapping. (A) Cluster distribution across study samples. Only patients with complete interkingdom datasets (pre‐ and postoperative 16S rRNA gene and ITS2 sequencing) were included in this analysis (*n* = 44). Left panel: dot plot displaying the relative abundance of each cluster (1–18) across all samples, where dot size represents cluster abundance and colors distinguish individual clusters. Right panel: stacked bar chart showing the proportional distribution of all 18 clusters within each sample, revealing sample‐specific community composition patterns. (B) KEGG metabolic pathway enrichment analysis across clusters. Heatmap displaying differential pathway enrichment patterns, where rows represent metabolic pathways organized by functional category and columns represent individual clusters (1–18). Color intensity indicates the magnitude of pathway enrichment (mean log_2_ fold change) when comparing samples with high versus low cluster abundance. White regions indicate no significant enrichment. The figure was generated using R.

To understand the functional implications of the identified microbial clusters, we performed comprehensive analysis of cluster distribution, abundance, and metabolic pathway mapping across all 18 clusters identified through network analysis (Figure 5).

#### 2.5.1 Preoperative Microbial Cluster Distribution and Heterogeneity

Analysis of cluster distribution across study samples revealed substantial heterogeneity in microbial community composition (Figure 5A). The analysis showed that cluster abundances varied vastly between individual samples, with some clusters (particularly clusters 1, 7, and 14) showing high abundance in multiple samples, while others (clusters 10, 13, and 18) appeared more sporadically and at lower abundances. No single cluster dominated across all samples, indicating a highly personalized gut microbiome landscape even within our relatively homogeneous surgical cohort.

Notably, the cluster distribution patterns revealed that most samples contained multiple active clusters simultaneously, with individual samples typically harboring 14–17 detectable clusters. This multi‐cluster architecture supports the idea that gut microbiome function emerges from the coordinated activity of multiple microbial subcommunities rather than from single dominant populations.

#### 2.5.2 Cluster Abundance and Functional Patterns

The prevalence analysis (Figure S6) identified cluster 7 as the most frequently predominant cluster, representing the primary cluster in 18.2% of samples, followed by clusters 11 (13.6%) and 14 (9.1%). Conversely, clusters such as 13, 15, 16, and 18 did not achieve abundance dominance in any of the samples. This dominance hierarchy suggests that certain microbial clusters possess competitive advantages or occupy more fundamental ecological niches within the gut ecosystem. The observation that even the most prevalent cluster (cluster 7) dominated in less than 20% of samples underscores the remarkable diversity of microbiome community states across the cohort. This heterogeneity may reflect differences in host genetics, diet, medication history, or other environmental factors that shape microbiota assembly.

#### 2.5.3 Taxonomic‐functional and Cluster Mapping Coverage

Next, we performed functional KEGG annotation mapping of the preoperative 16S and ITS2 data. Out of 7816 total ASVs identified in our dataset, 1572 (20%) were successfully identified on species‐level suitable for KEGG functional analysis, while the overall mapping success rate including genus level was 37.1% (1991 genus‐level ASVs). Functional mapping was achieved for 912 ASVs (representing 58% of all species‐identified ASVs in the dataset. These mapped ASVs corresponded to 124 unique KEGG organisms, from which 12,652 metabolic pathways were retrieved and analyzed across the microbial clusters.

For cluster analysis, 227 ASVs (80% mapping coverage) out of 283 ASVs assigned to network clusters by Louvain clustering had both KEGG mapping and cluster assignment and were included in the functional analysis.

#### 2.5.4 KEGG Pathway Enrichment Analysis

Differential pathway analysis identified significant enrichment patterns across clusters. The KEGG pathway enrichment heatmap (Figure 5B) revealed cluster‐specific metabolic signatures spanning multiple functional categories. Clusters 1 and 4 displayed extensive metabolic profiles with strong enrichment across degradation pathways (flavonoids, xylene, and aromatic compounds) and biosynthetic processes. Cluster 2 showed notable enrichment in steroid degradation and biosynthesis of siderophore group non‐ribosomal peptides, while cluster 3 demonstrated strong activity in nitrotoluene degradation. The diverse metabolic architecture across clusters indicates that gut resilience depends on both broad functional diversity and specialized metabolic roles, with different bacterial populations contributing distinct capabilities for xenobiotic metabolism, secondary metabolite production, and inter‐bacterial communication systems.

### 2.6 Network Hub Functional Profile

Cluster 15 showed no dominant abundance patterns across patients but significant associations with overall fungal diversity (*r* = 0.46). Functional analysis revealed that Cluster 15 possesses metabolic capabilities, including specialized functions like bacterial chemotaxis and nitrogen cycle processes. This suggests Cluster 15 serves a dual role of maintaining interkingdom network stability through fungal interactions while simultaneously contributing specific metabolic functions that may be crucial for microbiome resilience and adaptation to environmental perturbations, despite its relatively low abundance.

## 3 Discussion

Our study is one of the first human studies to examine the alteration of both bacterial and fungal gut microbiota after colorectal surgery in depth. With our data, we were able to investigate postsurgical fungal and bacterial microbiota dynamics and other clinical factors influencing postsurgical outcomes and gut microbiota interactions in the context of surgery. The strength of our study is the standard perioperative concept, which makes the results of the patients more comparable. Our findings demonstrate a significant reduction in bacterial gut diversity on the fifth/sixth postoperative day, while fungal diversity remains stable and thereby challenges the prevailing assumption that surgical stress uniformly disrupts all gut microbiota.

Our findings of significant bacterial diversity reduction align with previous studies suggesting that surgical stress is a critical factor in altering the microbiome's homeostasis [19, 27]. A 2022 meta‐analysis on microbiota diversity alterations in the context of gastrointestinal surgery (81% bariatric surgery) reported an association with increased postoperative alpha diversity and a shift in beta diversity, which may appear contrary to our findings at first glance [21]. However, this is likely attributable to the distinct baseline microbiota composition in bariatric patients, who often present with obesity‐associated dysbiosis [35]. Therefore, it is important to distinguish between bariatric and non‐bariatric patient populations when interpreting microbiota‐related surgical outcomes. Notably, studies involving patient populations more comparable to ours report similar findings, supporting the validity of our results. Schmitt et al. examined the gut microbiota of patients who underwent colorectal surgery preoperatively and 6, 9, and 12 months after the operation [23]. Postoperatively, there was a decrease in alpha diversity, which was still visible 12 months after the operation. Žukauskaitė et al. showed significant alterations of the beta‐diversity at 6 and 30 days after colorectal surgery, but the alpha diversity decreased slightly after surgery [36]. Fang et al. reported a significant decrease in species and metabolite diversity and a lasting microbial destabilization after surgery in patients with inflammatory bowel disease (IBD) [37]. In a small cohort of our own patients with Crohn's disease, who underwent colorectal resection, we were unable to detect any postoperative changes in the bacterial and fungal composition, which we attributed to the strongly dysbiotic microbiota that was already present preoperatively [38]. Because patients with IBD may have dysbiosis, we decided not to include them in the current study [39]. It is also important to consider that the composition of the gut microbiota can be influenced by a variety of factors, including ethnicity, geography, lifestyle, and dietary habits [40, 41]. As such, studies from Western countries are often difficult to compare with those from Asian countries, where these variables differ significantly. To gain a more comprehensive understanding of the role of the gut microbiota in surgical contexts, further studies across diverse populations and regions are essential.

Interestingly, we did not observe statistically significant bacterial alpha diversity differences in the preoperative microbiome in patients who had received presurgical antibiotic treatment (4‐week presurgical timeframe). However, we cannot assess the extent to which our postoperative findings of decreased bacterial alpha diversity in response to bowel resection surgery were confounded by the standard‐intraoperative antibiotic treatment with cephalosporins and nitroimidazoles. According to current recommendations, it is not possible to dispense with intraoperative antibiotic administration, since the aim of a single intraoperative dose is to prevent the occurrence of infectious complications such as surgical site infections [42]. We tried to make the patients in our study comparable by only including individuals whose operations were part of a standard perioperative protocol and also received the same intraoperative antibiotic treatment. It should be noted that in our study, no preoperative antibiotic bowel preparation was performed, as it is now recommended by the POMGAT guidelines [20]. Future studies will need to investigate this aspect, as such interventions are likely to have a significant impact on the composition and dynamics of the gut microbiome.

Interestingly, we observed no significant changes in fungal alpha and beta diversity in postoperative fecal samples, compared to the preoperative state. In contrast to bacterial diversity, fungal diversity remained relatively stable after surgery, except for subgroup‐specific trends. This resilience of the mycobiome suggests it may play a stabilizing role in maintaining gut homeostasis during periods of significant stress, such as surgery. This stability of the mycobiome contrasts sharply with the significant reduction seen in bacterial diversity following surgery. Several factors may explain this differential response. Fungi may possess greater resilience to surgical intervention than bacterial communities due to differences in growth rates, colonization strategies, or reduced susceptibility to perioperative antibiotics. The cell wall structure of fungi provides enhanced resistance to environmental stressors compared to bacterial cell membranes [43]. Additionally, many fungi can shift between different morphological forms (dimorphism), potentially enabling adaptation to changing gut conditions during the perioperative period. This phenomenon of mycobiome stability could play a critical role in maintaining certain gut functions when bacterial communities are disrupted, possibly contributing to recovery processes. The observed surgery‐specific trends in fungal diversity—decreasing after resection but increasing after continuity restoration—further suggest that surgical approach may differentially impact fungal communities, a finding that warrants further investigation for its potential clinical implications.

To our knowledge, this is the first study reporting the possible effects of colorectal surgery on both fungal and bacterial gut microbiota. This allowed us to assess fungal and bacterial microbiota alterations in a more interdependent context, which we further assessed by applying statistical interkingdom correlation and network analyses. The observed stability of fungal communities despite significant bacterial disruption suggests potential compensatory mechanisms within the gut ecosystem. This relative resilience of the mycobiome may play an important role in maintaining gut function during the postoperative period, when bacterial communities are disturbed. Commensal fungi may help to (i) preserve epithelial barrier integrity (for example, by influencing tight‐junctions and mucosal homeostasis), (ii) modulate immune responses at the mucosal surface, and (iii) promote colonization resistance, both indirectly through microbiota‐immune crosstalk and directly via interkingdom competition that shapes bacterial recolonization after perturbation [44, 45, 46]. The extensive interkingdom interactions we identified through network analysis (18 distinct clusters) highlight the interconnected nature of the gut ecosystem, where changes in one kingdom may have cascading effects on the other. These interactions may be particularly relevant for understanding postoperative recovery processes and could potentially be targeted in future interventions aimed at maintaining gut homeostasis during surgical stress.

Our comprehensive cluster‐based analysis with pathway mapping revealed substantial heterogeneity in preoperative microbiome community structure, with 18 distinct microbial clusters showing varied abundance patterns across patients. This personalized microbiome architecture, where no single cluster dominated all samples, demonstrates remarkable individual variation even within our homogeneous surgical cohort. The dominance hierarchy—with cluster 7 most abundant in 18.2% of samples followed by clusters 1 and 14 ‐ suggests that certain microbial communities occupy fundamental ecological niches or possess competitive advantages in the gut environment. Functional analysis revealed distinct metabolic roles across clusters, with clusters 1 and 4 showing the highest functional diversity and potential implications for surgical resilience. Notably, *Firmicutes*‐enriched communities often include butyrate‐producing taxa that are frequently associated with epithelial barrier support and anti‐inflammatory effects [47, 48]. However, our sequencing‐based inference cannot determine whether these pathways were functionally active or causal for postoperative outcomes. The central network hub (cluster 15) demonstrated limited metabolic specialization, with enrichment only in nitrogen cycle processes and bacterial chemotaxis, indicating its primary role as a structural coordinator with selective functional contributions. Clusters 2 and 3 showed specialized enrichment in steroid degradation and nitrotoluene degradation respectively. Several clusters demonstrated functional involvement in antimicrobial resistance and biosynthetic pathways. The enrichment of bacterial secretion systems and quorum sensing pathways across multiple clusters provides potential mechanistic insight into inter‐bacterial communication and coordination.

This multi‐cluster architecture reveals that gut microbiome resilience emerges from functional redundancy across specialized subcommunities rather than dependence on single dominant populations. The significant correlation between hub cluster abundance and fungal diversity (*r* = 0.46) suggests this architecture enables fungal stability to compensate for bacterial disruption, with fungal components maintaining essential functions when bacterial partners are compromised. Functional cluster profiles could inform risk stratification and guide targeted interventions to optimize surgical outcomes, with clusters enriched in epithelial interaction pathways potentially predicting postoperative wound healing capacity and infection risk.

Overall, the combination of metabolically specialized clusters and structural network coordinators may explain why some patients maintain better gut function during the perioperative period, suggesting that baseline microbiome analyses could serve as a predictor of surgical outcomes. Nevertheless, it should be emphasized that our study is exploratory in nature, with a relatively small cohort, and the findings regarding interkingdom interactions and cluster structures are intended to generate hypotheses rather than provide definitive conclusions. Future studies with larger patient populations will be required to validate these patterns and assess their potential clinical relevance.

Our study revealed significant shifts in community composition that may have functional implications. The decrease in beneficial bacteria such as *Faecalibacterium* and *Bifidobacterium* postoperatively is particularly concerning, as these genera are associated with anti‐inflammatory effects and production of short‐chain fatty acids that support gut barrier integrity. The concurrent increase in potentially pathogenic *Enterobacteriaceae* family members, including *Klebsiella* and *Enterobacter*, suggests a shift toward a potentially pro‐inflammatory gut environment that could influence wound healing and recovery [49]. Importantly, the stability of the fungal component despite these bacterial changes suggests that therapeutic approaches targeting beneficial fungi might help maintain gut homeostasis during periods when bacterial communities are disrupted. Our network analysis further supports this notion, demonstrating extensive connections between bacterial and fungal communities that could be leveraged to develop targeted microbial interventions in the perioperative setting.

Our findings indicate that preoperative microbiome profiling reveals substantial interindividual variation and functionally distinct microbial clusters, which may in future help to identify patients requiring enhanced perioperative support. The observed surgery‐specific trends on fungal diversity (decreased after resection, increased after continuity restoration) imply that surgical approach influences microbiome recovery patterns, potentially informing procedural selection and postoperative management strategies.

While our study provides valuable insights into postoperative gut microbiota alterations limitations should be acknowledged. The relatively small cohort size and the exploratory nature of the identified microbial clusters limit the statistical power of subgroup analyses. The collection of fecal samples at only two distinct time points (preoperative and 5–6 days postoperatively) limits our ability to track the temporal dynamics of microbial recovery. A more granular timeline with additional collection points would better characterize the trajectory of community changes. Moreover, our study focused on short‐term postoperative changes and cannot address the long‐term stability or recovery of the gut microbiome following colorectal surgery.

Additionally, the sample size, although sufficient for preliminary conclusions, could have been expanded under different circumstances. Unfortunately, the COVID‐19 pandemic imposed unforeseen challenges, including stricter hygiene protocols and limited patient interactions, which ultimately hindered further recruitment. Future studies incorporating longer follow‐up periods and integrating multi‐omic approaches would address these limitations and build upon our findings. Long‐term applications of perioperative gut microbiome analysis may include routine preoperative microbiome screening, personalized perioperative protocols based on baseline functional profiles and targeted interventions for high‐risk microbiome patterns.

In conclusion, this study demonstrates that bacterial and fungal gut communities exhibit fundamentally different responses to surgical stress after colorectal surgery, with fungi showing remarkable resilience that may serve compensatory functions during bacterial disruption. The identification of functionally diverse microbial clusters and extensive interkingdom networks provides a mechanistic framework for understanding gut ecosystem stability and suggests novel risk stratification methods and potential therapeutic targets for optimizing surgical outcomes.

These findings challenge current assumptions about uniform microbiome disruption and highlight the importance of considering the entire gut ecosystem—not just bacterial components—in perioperative care. The stability of fungal communities during bacterial instability represents both a fundamental biological insight and a potential therapeutic opportunity that warrants further investigation and clinical translation.

## 4 Materials and Methods

We conducted a prospective monocentric cohort study at the Department for General and Visceral Surgery, University Medical Centre Freiburg, Germany from April 2018 to August 2020. Before onset, the study was approved by the local ethical committee (EK‐FR: 535/17) and registered at the German Clinical Trial register (DRKS00014059) prior to patient recruitment.

### 4.1 Course of the Study and Captured Data

All eligible patients were informed about the study's purpose on the day before surgery and provided written informed consent to participate in line with ICH‐GCP and the declaration of Helsinki of October 2013. The intake of antibiotics and other medication, dietary habits, consumption of alcohol, cigarettes, or drugs, as well as the current illness, pre‐existing illnesses, and previous surgeries were documented. Study staff visited all patients on the third, sixth and nineth day after surgery to monitor their postoperative course. Clinical parameters of interest included nausea (10‐point scale) and vomiting (yes or no), fever (yes or no), analgesia (type of analgesia, according to the perioperative standard treatment), type of other medication, especially of antibiotics and laxatives, as well as the current diet. Stool samples were collected preoperatively and on the fifth to sixth postoperative day, depending on bowel movements. The samples were frozen at ‐80°C immediately after receipt until further processing. Clinical values and laboratory parameters were captured for later analysis. Patients were observed until postoperative Day 9 or discharge.

### 4.2 Inclusion and Exclusion Criteria

Eligible for inclusion were adult patients (≥18 years of age) of both sexes who had undergone elective colorectal surgery. Patients were required to be mentally capable of giving written informed consent. Eligible types of surgery were right or left hemicolectomy, ileocecal resection, resection of the sigmoid, rectal resection or the restoration of colonic continuity after previous discontinuity resection. Due to the effects of IBD on gut microbiota, no patients with IBD were included [38]. Emergency surgery and patients with ostomies were not considered. Patients who received an unplanned ostomy during surgery were excluded from the study.

### 4.3 Perioperative Standard Treatment

The standard treatment followed a postoperative fast‐track protocol, including early mobilization, adequate analgesia, shortened fasting, and the avoidance of drains, urinary catheters, and nasogastric tubes, in accordance with Schwenk's recommendations for fast‐track care [50]. All patients received intraoperative antibiotic prophylaxis with 2000 mg cefazolin and 500 mg metronidazole. Analgesia was provided by an epidural anesthesia with ropivacaine (0.2%) and sufentanil (25 µg), and an additional oral therapy with metamizole (1 g, four times a day). After removal of sufentanil treatment, patients received an oral dose of oxycodone (10 to 20 mg, twice a day), if necessary.

Long‐term medications were continued postoperatively unless contraindicated. Postoperative standard diet started with water and tea a few hours after surgery. In the evening of the surgery day and on the first postoperative day, patients were allowed to eat soup, mush, porridge and yoghurt. Subsequently, the patients received easily digestible food such as noodles, rice, rusk, smashed potatoes, steamed vegetables and white bread with jam on the second to third postoperative day. The return to a normal diet took place from the fourth postoperative day. Any deviation from the standard concept was documented.

### 4.4 16S rRNA and ITS2 Genes Amplicon Library Construction and Sequencing

Fecal bacterial and fungal deoxyribonucleic acid (DNA) extraction was conducted using the ZymoBIOMICS DNA Mini Kit (Zymo Research, Irvine, CA) with additional bead beating on a FastPrep‐24 homogenizer (MPBiomedicals, Santa Ana, CA). For bacterial sequencing, we targeted the V3‐4 hypervariable region of the 16S ribosomal ribonucleic acid (rRNA) gene to construct libraries with dual indexing using the primer pair 341F (5′‐CCT ACG GGN GGC WGC AG‐3′) and 805R (5′‐GAC TAC HVG GGT ATC TAA TCC‐3′) as described by Klindworth et al. [51]. The polymerase chain reaction (PCR) protocol consisted of 20 cycles for bacterial DNA amplification followed by 10 cycles for subsequent biochemical barcode addition. The final library was sequenced using the MiSeq v2 reagent kit (500 cycles) (Illumina Inc., San Diego, CA, USA).

For fungal analysis, Internal Transcribed Space region 2 (ITS2) gene libraries were constructed following the dual indexing strategy of Kozich et al., using the gITS7 (forward: GTGARTCATCGARTCTTTG) primer described by Ihrmark et al. and the ITS4ngs (reverse: TTCCTSCGCTTATTGAT) primer [52, 53]. These ATGC primers anneal to the 5.8S and large subunit (LSU) rRNA genes flanking the ITS2 region. The PCR program included an initial denaturing step at 98°C for 30 s, followed by 40 cycles of 9 s at 98°C, 30 s at 56°C, and 30 s at 72°C, with final extension at 72°C for 10 min using Phusion Hot Start II DNA High‐Fidelity DNA Polymerase. For quality control, we included negative controls and a standard bacterial and fungal mock community (Zymo Research, Irvine, CA) as positive controls in all PCRs and sequencing runs. PCR products were purified enzymatically, and barcodes containing Illumina sequencing adapters were added in a second PCR reaction using the Quick‐16S NGS Library Prep Kit (Zymo Research, Irvine, CA).

PCR products were quantified on a 1.5% agarose gel and using a Qubit 4.0 fluorometer (Thermo Fisher Scientific, Waltham, MA, USA), then pooled to generate equimolar subpools. When necessary, the final pooled library was purified with the Select‐a‐Size DNA Clean & Concentrator (Zymo Research, Irvine, CA). Libraries were quantified using a NEBNext library quantification kit (New England BioLabs GmbH, Frankfurt am Main, Germany) and analyzed on a QiaXcel advanced system (Qiagen, Hilden, Germany) before sequencing on a MiSeq system using the MiSeq v2 reagent kit (500 cycles) with a 20% PhiX spike‐in.

### 4.5 Sequencing Data Processing and Statistical Analysis

Raw FASTQ files were initially evaluated for quality using FastQC. Subsequent quality control, trimming, and analysis of short reads were performed using the DADA2 pipeline, with visualization facilitated by multiple R packages within a Linux environment. ASVs extracted from DADA2 were taxonomically classified using the UNITE database for fungi and the Genome Taxonomy Database (GTDB) database for bacteria [54, 55].

Analysis of interactions between mycobiome and bacteriome were performed by comparing the relative abundances of bacterial and fungal taxa using the Spearman method with the corrplot package in R. For diversity analyses, alpha diversity Shannon and Inverse Simpson indices were calculated and normality was assessed using Shapiro–Wilk tests. Bacterial beta diversity was assessed using non‐metric multidimensional scaling (NMDS) with Bray–Curtis dissimilarity distances as the measurement metric. Statistical significance of perioperative differential abundances in alpha and beta diversity was evaluated using Wilcoxon signed‐rank tests for paired samples and Adonis (PERMANOVA) testing, respectively. LDA effect size analysis (LEfSe) was conducted to identify bacterial and fungal genera that function as discriminative features, enabling the distinction between classes (pre‐/post‐surgical samples) with a LDA score threshold ≥3.5 [56, 57].

Network construction and cluster identification were performed using the SpiecEasi R package (version 1.1.3) with Louvain community detection algorithm to identify microbial clusters [56]. This approach allows identification of meaningful associations while minimizing spurious correlations common in compositional data. To identify potential clinical drivers of microbiota changes, sequencing data were correlated with clinical parameters using appropriate statistical methods including the Benjamini‐Hochberg method (FDR <0.05) in R programming language.

Functional pathway analysis was performed using KEGGREST package (v1.32.0) to predict metabolic potential based on cluster and taxonomic profiles. Kyoto Encyclopedia of Genes and Genomes (KEGG) pathway analysis was performed by identifying organisms with absolute log_2_ fold change > 1.0 between high and low cluster abundance samples. Pathway enrichment was calculated as the mean log_2_ fold change of constituent organisms within each pathway. All analyses were performed in R v4.5.0. and v4.3.1. Figures were generated by R (Figures 2, 3, 4, 5 and Figures of Supporting Information) and Microsoft PowerPoint (Figure 1). Statistical significance was defined as *p* < 0.05 for all tests unless otherwise specified.

## Author Contributions

R.H. and A.K.L. designed the study. A.M., A.K., L.K., and A.K.L. were responsible for patient recruitment and data collection. S.W., E.K., CK., M.T.B., and A.K.L. performed the statistical analysis. R.H. and L.K. supervised the overall conduct of the study and provided critical guidance during all phases. E.K. and C.K. performed quality control. S.W., E.K., R.H., C.K., M.T.B., and A.K.L. interpreted the results and drafted the manuscript. All authors have read and approved the final manuscript.

## Funding

This study did not receive any specific funding. Dr. A.‐K. Lederer is supported by the Clinician Scientist Fellowship “Else Kröner Research College: 2022_EKFK.05”.

## Ethics Approval

Ethical approval for this study was obtained from the Ethics Committee of the Albert Ludwig University of Freiburg, Germany (EK‐FR: 535/17).

## Conflicts of Interest

The authors declare no conflict of interest.

## Supporting information




**Figure S1**: Error rates of 16S and ITS2 sequencing data.
**Figure S2**: Bacterial beta diversity in patients before and after surgery measured by NMDS. Mapping of pre‐ and postoperative bacterial beta diversity with clinical metadata.
**Figure S3**: LEfSe classification identifying bacterial genera that differentiates between pre‐ and postoperative states.
**Figure S4**: Surgery‐specific effects on fungal alpha diversity measured by Shannon index, stratified by surgical procedure.
**Figure S5**: Network representation on kingdom level, network interconnectivity analysis and Louvain cluster bar plot.
**Figure S6**: Predominance cluster analysis.

## Data Availability

Clinical data are available from the corresponding author on reasonable request. Due to local ethical regulations regarding data protection, the data cannot be made open. Data deposition: PRJEB90497
