# Supplementary material for: Divergent Resilience of Bacterial and Fungal Gut Microbiota After Colorectal Surgery: Insights From a Prospective Longitudinal Cohort Study
Source: MedComm (2020). 2026 May 26;7(6):e70781. doi: 10.1002/mco2.70781 (PMC13239092; doi:10.1002/mco2.70781)
Supplement: Supplementary file 1 — Figure S1: Error rates of 16S and ITS2 sequencing data. Figure S2: Bacterial beta diversity in patients before and after surgery measured by NMDS. Mapping of pre‐ and postoperative bacterial beta diversity with clinical metadata. Figure S3: LEfSe classification identifying bacterial genera that differentiates between pre‐ and postoperative states. Figure S4: Surgery‐specific effects on fungal alpha diversity measured by Shannon index, stratified by surgical procedure. Figure S5: Network representation on kingdom level, network interconnectivity analysis and Louvain cluster bar plot. Figure S6: Predominance cluster analysis. [file MCO2-7-e70781-s001.docx]

Divergent Resilience of Bacterial and Fungal Gut Microbiota after Colorectal Surgery: Insights from a prospective longitudinal cohort study

Short running title: resilience of gut microbiota after surgery

Simon Wetzel^1,2,3^, Eva Kohnert^4^, Roman Huber^5^, Alexander Müller^5^, Agnes Knott^5^, Lampros Kousoulas^6^, Clemens Kreutz^4^, Mohamed Tarek Badr^1,7,#^ , Ann-Kathrin Lederer^5,8,#,*^

1: Institute of Medical Microbiology and Hygiene, Medical Centre - University of Freiburg, Faculty of Medicine, University of Freiburg, Freiburg, Germany

2: Centre for Inherited Metabolic Diseases (CMMS), Karolinska University Hospital, Solna, Sweden

3: Department for Medical Biochemistry and Biophysics, Karolinska Institute, Stockholm, Sweden

4: Institute of Medical Biometry and Statistics, Faculty of Medicine and Medical Centre, University of Freiburg, 79104 Freiburg, Germany

5: Centre for Complementary Medicine, Department of Medicine II, Medical Centre - University of Freiburg, Faculty of Medicine, University of Freiburg, Freiburg, Germany

6: Department of General and Visceral Surgery, Medical Centre - University of Freiburg, Faculty of Medicine, University of Freiburg, Freiburg, Germany

7: Institute of Medical Microbiology and Hospital Hygiene, Medical Faculty, Otto von Guericke University Magdeburg, Magdeburg, Germany

8: Research Group Integrative Medicine, Department of General and Visceral Surgery, University Hospital Ulm, Ulm, Germany

^#^ These authors co-supervised this work

*Corresponding author:

PD Dr. Ann-Kathrin Lederer
Centre for Complementary Medicine, Department of Medicine II, Medical Centre - University of Freiburg, Faculty of Medicine, University of Freiburg
Sir-Hans-A.-Krebs-Straße, 79106 Freiburg, Germany
ann-kathrin.lederer@uniklinik-freiburg.de
ORCID: 0000-0001-7984-9530

Supplementary Figure Legends

**
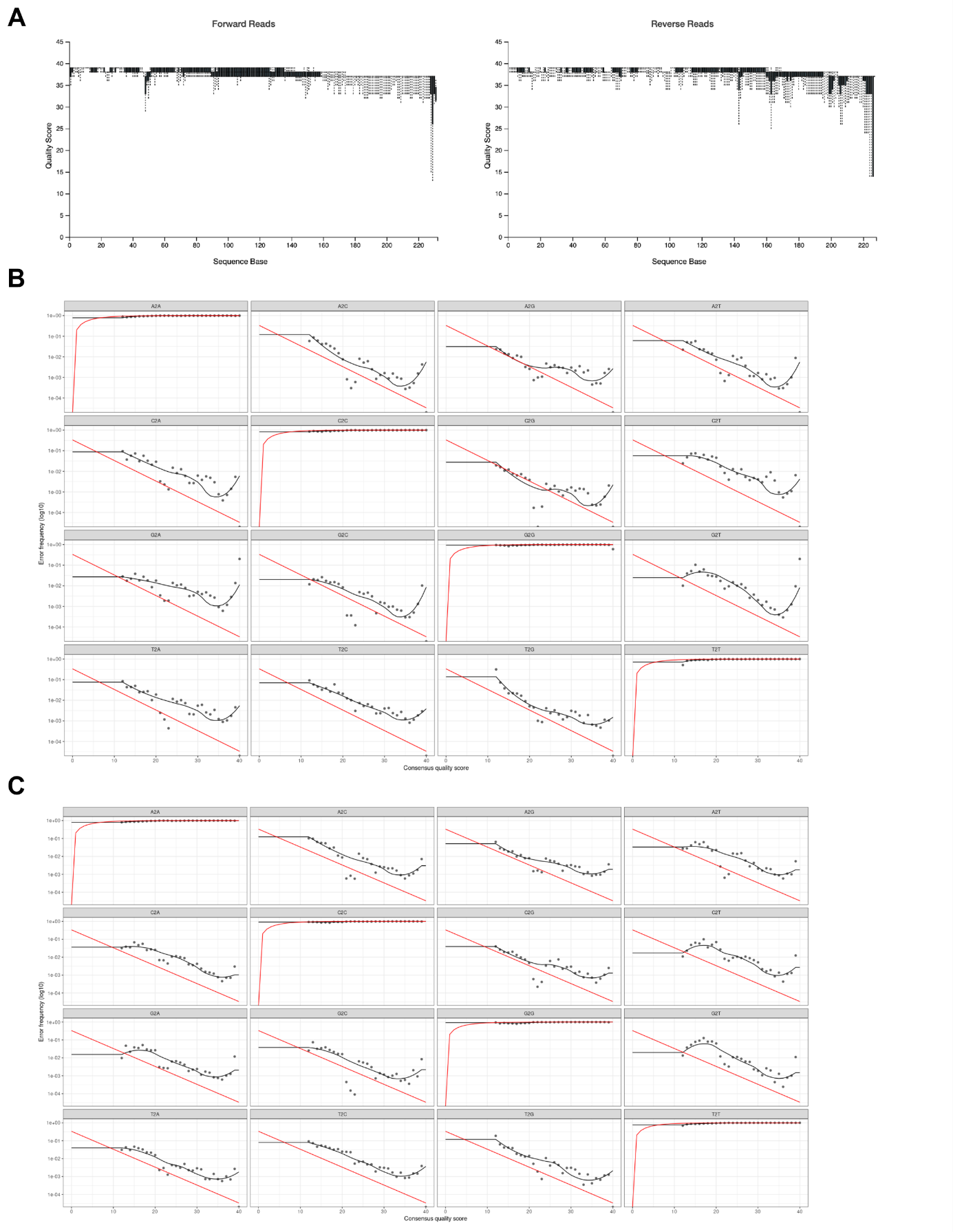
**

**Figure S1**: **Error rates of 16S and ITS2 sequencing data**. **(A)** Base calling error rates of bacterial 16S sequencing data (left panel: forward reads, right panel: reverse reads). **(B)** Base calling error rates of fungal ITS2 sequencing data (forward reads). Red line represents expected error frequency. **(B)** Base calling error rates of fungal ITS2 sequencing data (forward reads). Red line represents expected error frequency. The figure was generated using R.

**
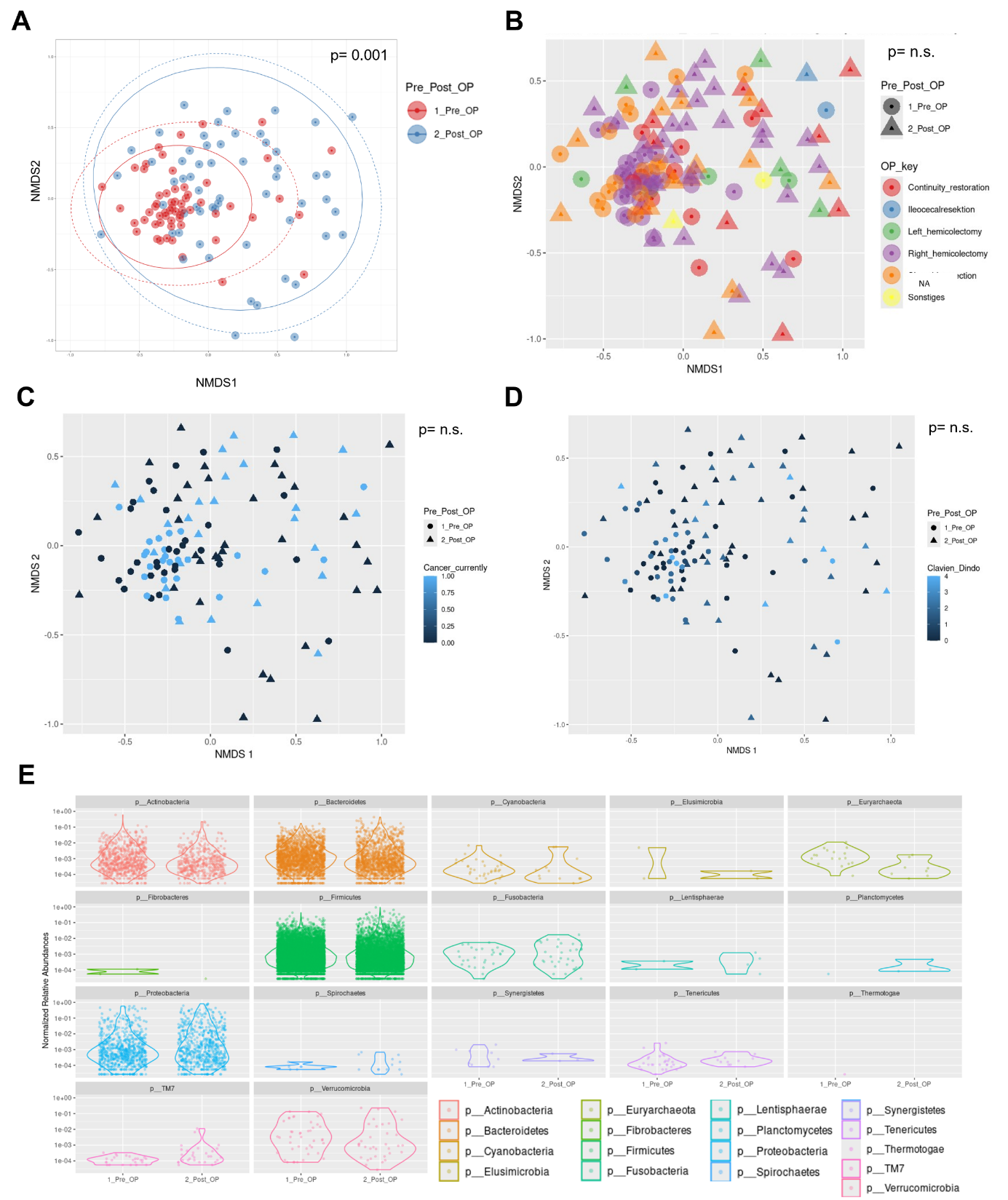
**

**Figure S2: (A)** Bacterial beta diversity in patients before and after surgery measured by NMDS. Mapping of pre- and postoperative bacterial beta diversity with clinical metadata, such as operational techniques **(B)**, cancer disease **(C)** or postoperative complications **(D)**, revealed no apparent driver for postoperative increase in beta-diversity. **(E)** Phyla with substantial abundance levels showed no significant differences in normalized relative abundance between pre- and postoperative samples. The figure was generated using R.

**
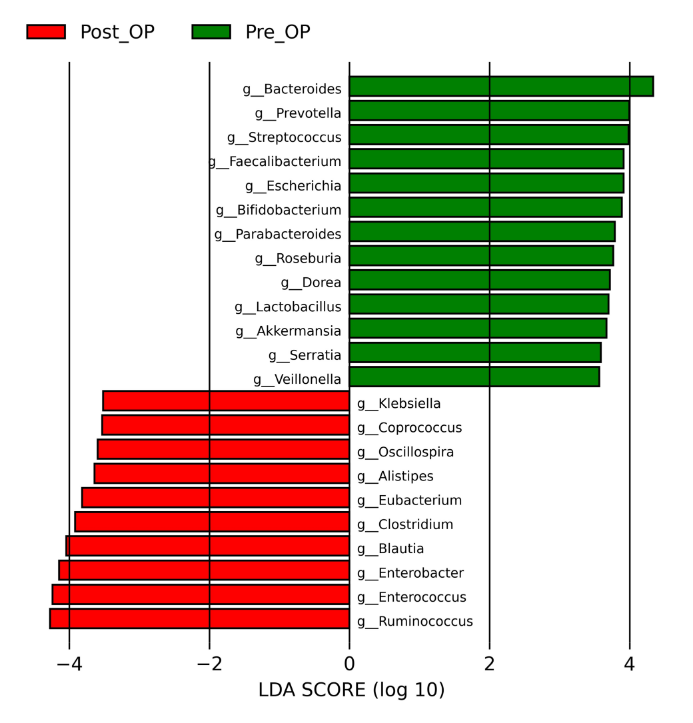
**

**Figure S3: (A)** LEfSe classification identifying bacterial genera that differentiates between pre- and postoperative states. Horizontal bars represent the LDA score (log 10 transformed) for each discriminative genus, with green bars indicating genera enriched preoperatively and red bars indicating genera enriched postoperatively. Only genera with high LDA score >3.5 are displayed. Pre_OP=preoperative, Post_OP=postoperative, g=taxonomic genus level. The figure was generated using R.

**
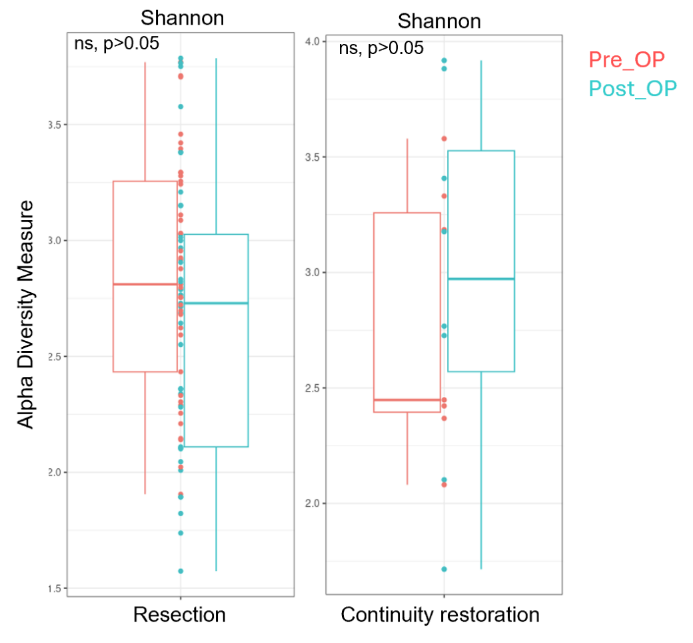
**

**Figure S4:** Surgery-specific effects on fungal alpha diversity measured by Shannon index, stratified by surgical procedure (p>0.05). Pre_OP=preoperative, Post_OP=postoperative. The figure was generated using R.

**
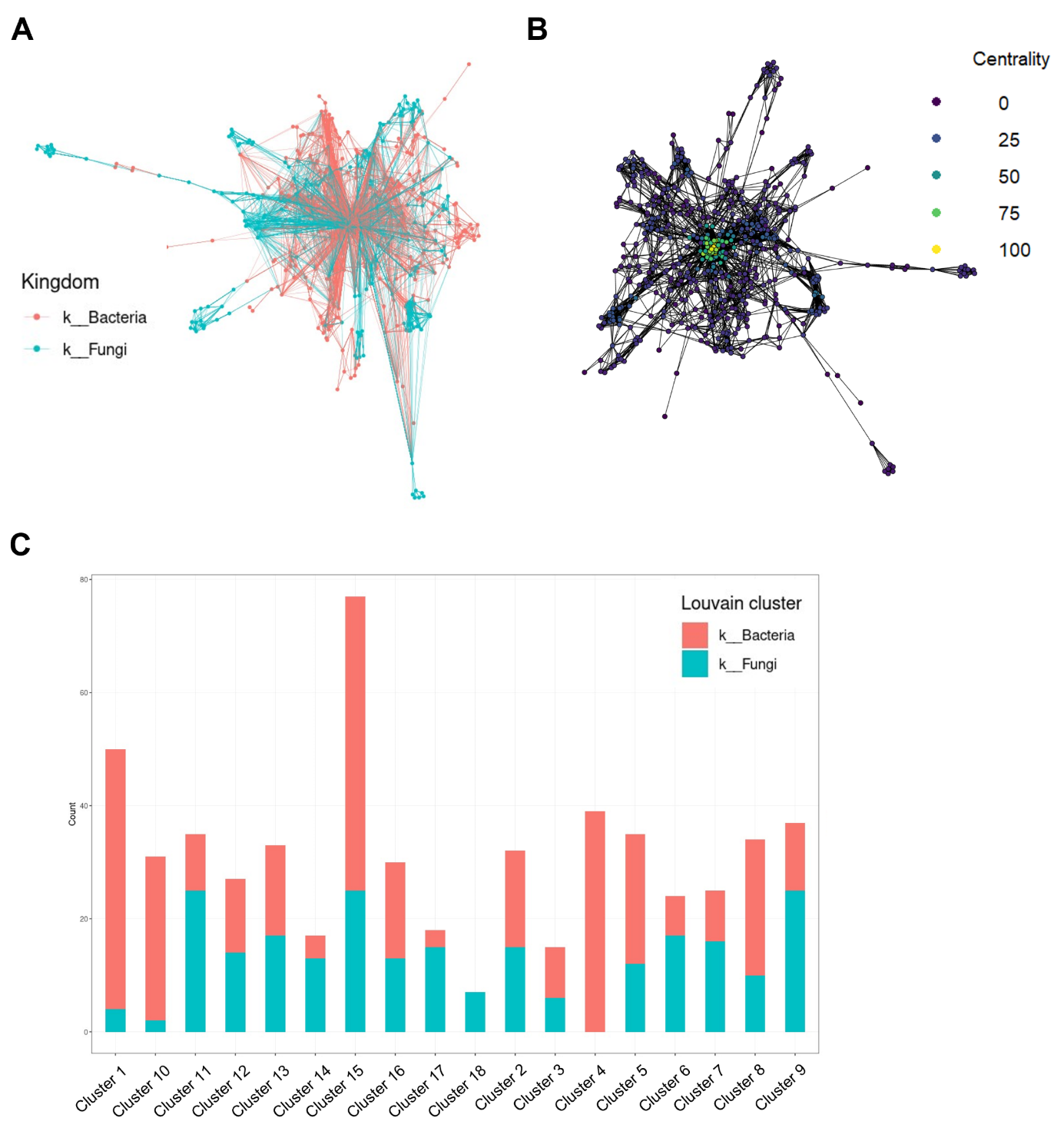
**

**Figure S5: (A)** Network representation on kingdom level reveals closely interconnected ASVs of bacterial and fungal origin in preoperative microbiota **(B)** Network interconnectivity analysis representing network degree values highlights central fungal and bacterial ASV-cluster of high interconnectivity **(C)** Louvain cluster bar plot with in total 18 microbiota clusters (16 clusters of both bacterial and fungal origin). Central cluster 15 composition with ~70% bacterial and ~30% fungal ASVs. The figure was generated using R.

**
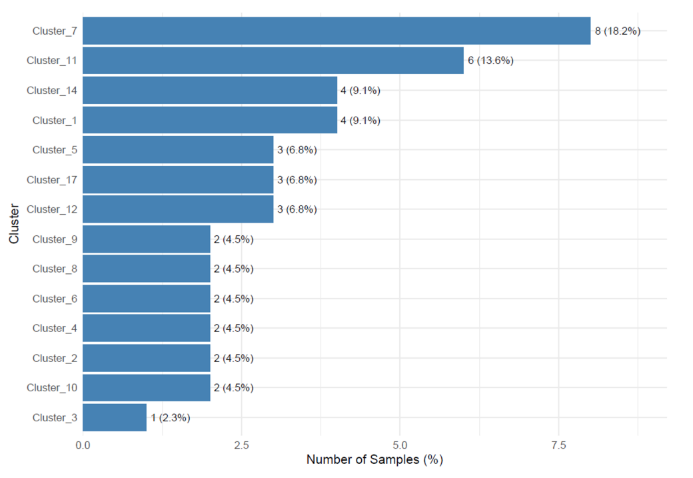
**

**Figure S6: Predominance cluster analysis.** Barplot representing most frequently found predominant clusters in patients’ preoperative microbiome. The figure was generated using R.
